# Modelling the Flow Behaviour of Al Alloy Sheets at Elevated Temperatures Using a Modified Zerilli–Armstrong Model and Phenomenological-Based Constitutive Models

**DOI:** 10.3390/ma17071584

**Published:** 2024-03-29

**Authors:** Ali Abd El-Aty, Yong Xu, Yong Hou, Shi-Hong Zhang, Sangyul Ha, Liangliang Xia, Bandar Alzahrani, Alamry Ali, Mohamed M. Z. Ahmed, Abdallah Shokry

**Affiliations:** 1 Department of Mechanical Engineering, College of Engineering at Al Kharj, Prince Sattam Bin Abdulaziz University, Al-Kharj 11942, Saudi Arabiamoh.ahmed@psau.edu.sa (M.M.Z.A.); 2 Mechanical Engineering Department, Faculty of Engineering, Helwan University, Cairo 11795, Egypt; 3 Shi-Changxu Innovation Center for Advanced Materials, Institute of Metal Research, Chinese Academy of Sciences, Shenyang 110016, China; 4 Department of Materials Science and Engineering & RIAM, Seoul National University, Seoul 08826, Republic of Korea; 5 Department of Semiconductor Engineering, Myongji University, Yongin 17058, Republic of Korea; dubuking@postech.ac.kr; 6 School of Transportation, Ludong University, Yantai 264025, China; 7 Department of Mechanical Engineering, Faculty of Engineering, Fayoum University, Fayoum 63514, Egypt

**Keywords:** flow behaviour, modified Zerilli–Armstrong, phenomenological constitutive models, physical-based constitutive models, strain rate, elevated temperatures

## Abstract

The flow behaviour of AA2060 Al alloy under warm/hot deformation conditions is complicated because of its dependency on strain rates ($\dot{\varepsilon }$), strain (ε), and deformation modes. Thus, it is crucial to reveal and predict the flow behaviours of this alloy at a wide range of temperatures (T) and $\dot{\varepsilon }$ using different constitutive models. Firstly, the isothermal tensile tests were carried out via a Gleeble-3800 thermomechanical simulator at a T range of 100, 200, 300, 400, and 500 °C and $\dot{\varepsilon }$ range of 0.01, 0.1, 1, and 10 s^−1^ to reveal the warm/hot flow behaviours of AA2060 alloy sheet. Consequently, three phenomenological-based constitutive models (L-MJC, S1-MJC, S2-MJC) and a modified Zerilli–Armstrong (MZA) model representing physically based constitutive models were developed to precisely predict the flow behaviour of AA2060 alloy sheet under a wide range of T and $\dot{\varepsilon }$. The predictability of the developed constitutive models was assessed and compared using various statistical parameters, including the correlation coefficient (*R*), average absolute relative error (*AARE*), and root mean square error (*RMSE*). By comparing the results determined from these models and those obtained from experimentations, and confirmed by *R*, *AARE*, and *RMSE* values, it is concluded that the predicted stresses determined from the S2-MJC model align closely with the experimental stresses, demonstrating a remarkable fit compared to the S1-MJC, L-MJC, and MZA models. This is because of the linking impact between softening, the strain rate, and strain hardening in the S2-MJC model. It is widely known that the dislocation process is affected by softening and strain rates. This is attributed to the interactions that occurred between ε and $\dot{\varepsilon }$ from one side and between ε, $\dot{\varepsilon }$, and T from the other side using an extensive set of constants correlating the constitutive components of dynamic recovery and softening mechanisms.

## 1. Introduction

Al alloys are eco-friendly metallic materials renowned for their distinctive characteristics, making them highly desirable across various sectors. Their low density plays a crucial role in decreasing weight, which is vital for improving fuel efficiency and performance in the automotive, aerospace, and aviation industries [1]. The considerable strength of Al alloys enables the production of robust and dependable components, a necessity in aerospace engineering. Their outstanding corrosion resistance extends the lifespan of products and minimizes the need for maintenance, particularly in challenging environments such as marine settings or the aerospace industry [2]. Al alloys are highly recyclable, fitting seamlessly into sustainable practices by allowing for their reuse and repurposing with a minimal loss of quality. This feature is precious in the context of modern environmental awareness, helping to lessen the consumption of resources and the environmental footprint [3]. Al alloys are commonly used in the automotive industry to manufacture engine blocks, frames, and body panels. In the aerospace and aviation industries, they are integral to constructing fuselage, wing structures, and other essential components, benefiting from their excellent strength-to-weight ratio. Al alloys’ adaptability and superior attributes make them essential for cutting-edge engineering and manufacturing [4,5].

One of the Al alloys that gained significant attention in the aircraft, military, and aerospace industries due to their outstanding mechanical and physical properties compared to conventional Al alloys is AA2060 [6]. This alloy is distinguished by its exceptional strength-to-weight ratio, high modulus of elasticity, and enhanced fatigue resistance, positioning it as a prime candidate for applications demanding weight efficiency and robust strength [7]. Furthermore, AA2060 demonstrates commendable corrosion resistance and the ability to endure extreme temperatures and environmental conditions, making it suitable for challenging applications. The remarkable attributes of AA2060 are attributed to the inclusion of Li, which significantly influences the modulus of elasticity and contributes to weight reduction [1,7]. Specifically, incorporating just 1% of Li into an Al alloy can decrease its density by roughly 3% and augment its modulus of elasticity by about 6%. This enhancement is attributed to the unique atomic and structural properties of Li, which fortify the Al matrix through the formation of a finely dispersed phase of Li-containing compounds [1,8]. Despite its outstanding mechanical and physical characteristics, including an exceptional strength-to-weight ratio and enhanced fatigue resistance, AA2060 encounters significant challenges with formability. It exhibits pronounced anisotropic behaviour, particularly at room temperature [9]. These issues have limited its industrial applications, as it can be difficult to form complex shapes using traditional cold-forming techniques [10]. As a result, alternative techniques, such as deformation at high strain rates and forming at elevated temperatures, are frequently employed to improve the formability of AA2060 and surmount its inherent drawbacks [9,10,11,12,13,14,15]. Consequently, a deep understanding of the deformation behaviour of the AA2060 alloy at elevated temperatures is vital to producing reliable components from this material. This requires experimental investigations to evaluate the mechanical behaviour of AA2060 under different T, $\dot{\varepsilon }$, and loading conditions as well as the use of advanced modelling approaches such as finite element analysis (FEA) to simulate and predict the deformation behaviour of the material [16].

The confidence in simulating the plastic deformation of a specific material using FE mainly relies upon the reliability and accuracy of the constitutive relations to describe the behaviour of this material, particularly when the material exhibits anisotropic behaviour [17,18,19]. Thus, several constitutive models were developed and proposed over the last few years to predict the flow behaviour of various metallic materials at elevated temperatures. These constitutive models include physically based constitutive models, phenomenological constitutive models, and artificial neural network-based modelling (ANN) [20,21,22,23,24]. The optimal constitutive model should possess a moderate number of material parameters, which may be assessed via a few experimental data, and accurately predict the mechanical behaviour of materials over a wide range of rheological variables [20,21].

Physically based constitutive models may afford exact representations of the flow behaviour of metallic materials over a wide range of rheological variables [23]. Furthermore, they can trace the microstructural evolution by using the dislocation density as a variable, in which the constitutive equations based on dislocation theory may correctly characterize the effects of strain hardening and dynamic softening [24,25,26,27]. Notable examples of physically based constitutive models include the Zerilli–Armstrong (ZA), Dynamic recrystallization, and the Preston–Tonks–Wallace models [28]. Specifically, the ZA model is designed to account for the synergistic effects of strain hardening, strain-rate hardening, and T softening on the flow behaviour of metals, making it highly regarded for its ability to predict the deformation behaviour of materials at high temperatures [29,30,31]. Samantaray et al. [32,33,34,35,36] modified the ZA model to also factor in thermal softening, enhanced strain-rate hardening, and isotropic hardening as well as the compound influence of $\dot{\varepsilon }$ and T on the flow stress. This modified ZA model has proven effective in precisely determining the high T flow behaviour of various metallic materials. Therefore, the modified ZA (MZA) constitutive model was considered in this study [32].

Phenomenological-based constitutive models simplify the prediction of material flow behaviour across a broad range of $\dot{\varepsilon }$ and T without necessitating a comprehensive understanding of the rheological factors involved in the forming process [37,38,39,40,41,42]. These models are primarily derived through empirical fitting and regression analysis, making them particularly useful for modelling materials’ flow behaviour and integrating with FE codes to replicate real-world forming processes under various conditions [43,44,45,46,47]. The Johnson–Cook (JC) model has gained popularity in various FE applications due to its fast computation speed, minimal computational demands, and straightforward formulation [48,49]. However, a significant limitation of the JC model is its assumption that the impacts of $\dot{\varepsilon }$ and T on flow stresses are independent, neglecting the interactive effects between these variables. This oversight can significantly diminish the model’s predictive accuracy and reliability. Consequently, numerous studies have sought to refine the JC model to better account for the coupled effects of $\dot{\varepsilon }$ and T. Despite improvements to the JC model, further enhancements are necessary to capture the coupled influences of $\dot{\varepsilon }$ and T on accurately predicting the flow behaviour of metals under different forming conditions.

Based on the discussion mentioned above, it is concluded that it is crucial to reveal and predict the flow behaviour of the AA2060 Al alloy at elevated temperatures using different constitutive models. This literature review identifies four constitutive models as effective in predicting the flow behaviours of metallic materials and capturing their non-linear behaviour, such as the modified ZA (MZA) model and three modified JC models proposed by Lin et al. [50] and Shokry et al. [51,52]. Consequently, this study aims to develop these four constitutive models to precisely predict the flow behaviour of AA2060 Al alloy under a wide range of T and $\dot{\varepsilon }$. The predictability of the developed constitutive models is assessed and compared using various statistical parameters, including the correlation coefficient (*R*), average absolute relative error (*AARE*), and root mean square error (*RMSE*).

## 2. Experimental Procedures

The material utilized in the current study was a rolled AA2060 Al alloy sheet. A Gleeble-3800 simulator was utilized to accomplish the isothermal tensile tests at T = 100, 200, 300, 400, and 500 °C and $\dot{\varepsilon }=0.01,\;0.1,\;1,\;and\;10$ s^−1^. To prevent overheating of the tensile samples, the heating process was divided into two stages. Initially, the sample was heated to a temperature 30 °C below the target deformation T at a rate of 20 °C/s. Subsequently, the final increment to the desired T was accomplished at a slower rate of 5 °C/s. The samples were then maintained at the set deformation T for 5 min to eliminate thermal gradients and ensure a uniform T distribution along the gauge length. After that, the tensile samples were stretched to fracture with a specified $\dot{\varepsilon }$ and then immediately quenched in the water to preserve the microstructures formed at high T. In order to ensure the reliability and consistency of results, every test condition was repeated five times across all test samples, and the average value was computed for each condition.

## 3. Result and Discussion

### 3.1. Flow Behaviour

**Figure 1:**
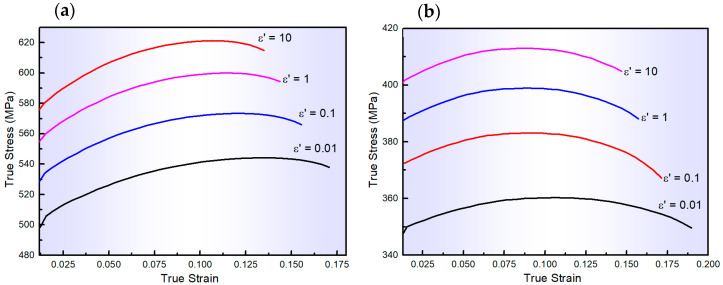

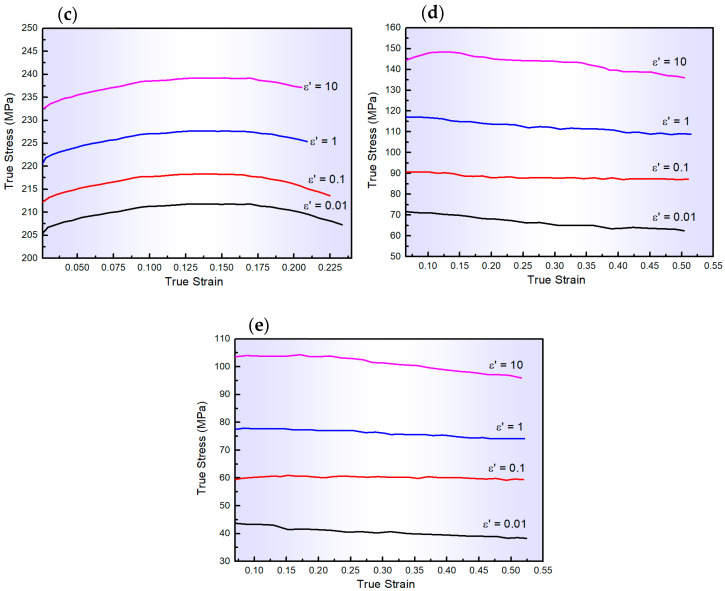
The flow curves of AA2060 sheets tested at the $\dot{\varepsilon }$ range of 0.01–10 s^−1^ and testing temperatures of (**a**) 100, (**b**) 200, (**c**) 300, (**d**) 400, and (**e**) 500 °C, respectively.

The flow curves depicted in Figure 1 were captured from the isothermal tensile test performed at the abovementioned testing conditions. As shown in Figure 1, the flow behaviours of AA2060-T8 are notably affected by both $\dot{\varepsilon }$ and T. As $\dot{\varepsilon }$ increases, the flow stresses also rise, while they decrease with higher T. Initially, during the early stages of deformation, there is a rapid increase in flow stresses due to extensive work hardening, overpowering any dynamic softening effects. However, as deformation progresses, dynamic softening mechanisms such as dynamic recovery appear, which can partially or wholly offset the influence of work hardening. At the ultimate tensile stress point, there is a balance between strain hardening and dynamic softening, resulting in a gradual decrease in flow stress or no significant change. Therefore, the impact of $\dot{\varepsilon }$ and T is substantial and should be adequately incorporated into constitutive modelling.

### 3.2. Constitutive Modelling

The appropriate constitutive models can effectively correlate the deformation parameters such as flow stress, ε, $\dot{\varepsilon }$ and T. Thus, constitutive modelling has been widely applied in flow behaviour prediction [31,32,33,34,35,36,37,38,39,40] and forming simulation [41,42,43] studies. Constitutive models are classified into physically based constitutive models [27,28,29,30,31,32,33,34,35,36], phenomenological constitutive models [37,38,39,40,41,42,43,44,45,46,47,48,49,50,51,52], and machine learning-based modelling [53,54,55]. The optimal constitutive model should possess a moderate number of material parameters, which may be assessed via a few experimental data, and accurately predict the mechanical behaviour of materials over a wide range of rheological variables [50,51,52,53]. In this study, the physically based MZA constitutive model and three phenomenological-based modified JC models proposed by Lin et al. [50] and Shokry et al. [51,52] were developed to precisely predict the flow behaviour of AA2060 Al alloy under a wide range of $\dot{\varepsilon }$ and T. The details of each constitutive model are discussed in this section.

#### 3.2.1. Modified ZA (MZA) Constitutive Model

The ZA model [34], a widely recognized physically based model, is formulated based on the principles of dislocation mechanisms that are crucial in the plastic deformation of metallic metals under various forming conditions. In the ZA model, the flow stress is divided into thermal and athermal components as written in Equation (1).

(1) $$\sigma =\sigma_{th}+\sigma_{a}$$

where $\sigma_{a}$ represents the athermal activation flow stress and $\sigma_{th}$ denotes the thermal activation flow stress.

Compared to other dislocation-based models, the ZA model features a relatively simple expression. A vital aspect of this model is that the expression varies for each type of material structure, reflecting the different $\dot{\varepsilon }$ controlling mechanisms specific to each structure. For body-centred cubic (BCC) and face-centred cubic (FCC) metallic materials, Equations (2) and (3) for the thermal activation flow stress are expressed as follows:

(2) $$\sigma_{th}=C_{1}exp(-C_{3}T+C_{4}T\;ln\dot{\varepsilon \;})$$

(3) $$\sigma_{th}=C_{2}\varepsilon^{1/2}(-C_{3}T+C_{4}T\;ln\dot{\varepsilon \;})$$

where Equations (2) and (3) are used for BCC and FCC metallic materials, respectively. $C_{1}$, $C_{2}$, $C_{3}$, and $C_{4}$ are material constants, and T represents the testing temperature.

By integrating the $\sigma_{th}$ with the influence of the yield stress on the grain size into a single component called $C_{0}$, two ZA models for BCC and FCC metallic materials are written as follows:

(4) $$\sigma ={C_{0}+C}_{1}exp(-C_{3}T+C_{4}T\;ln\dot{\varepsilon )+C_{5}\varepsilon^{n}}$$

(5) $$\sigma ={C_{0}+C}_{2}\varepsilon^{1/2}(-C_{3}T+C_{4}T\;ln\dot{\varepsilon \;})$$

where Equations (4) and (5) are used for BCC and FCC metallic materials, respectively. σ, and ε are the von Mises flow stress and equivalent plastic strain, respectively. ${\;C}_{5}$ and n are material constants. All these material constants need to be determined.

While the ZA constitutive model accounts for dislocation mechanisms, it overlooks the influence of deformation conditions, which differ from the practical forming process, thus diminishing its predictive accuracy. To address these limitations, Samantaray et al. [35] proposed an MZA model by coupling between softening and both ε and $\dot{\varepsilon }$ to effectively determine the flow behaviour of metallic materials at elevated forming temperatures. The MZA model proposed by Samantaray et al. [35] is written as follows:

(6) $$\sigma =\left(C_{1}+C_{2}\varepsilon^{n}\right)\exp\left[-\left(C_{3}+C_{4}\varepsilon \right)T^{*}+\left(C_{5}+C_{6}T^{*}\right)\ln\varepsilon^{˙*}\right]$$

where $\varepsilon^{˙*}$ is the ratio between the testing ($\dot{\varepsilon \;})$ and reference strain rates (${\dot{\varepsilon }}_{r})$, and $T^{*}$ is the difference between testing (T) and reference temperatures ($T_{r})$. $\varepsilon^{˙*}$ and $T^{*}$ are expressed as follows:

(7) $$\varepsilon^{˙*}=\frac{\dot{\varepsilon \;}}{{\dot{\varepsilon }}_{r}}$$

(8) $$T^{*}=T-T_{r}$$

As written in Equation (6), the MZA model comprehensively considered the influence of thermal softening, strain-rate hardening, and strain hardening on the flow behaviour of metals at elevated temperatures.

To determine the MZA model constants in this investigation, ${\dot{\varepsilon }}_{r}$ and $T_{r}$ are adjusted to be 0.01 s^−1^ and 100 °C, respectively. Thus, Equation (6) is reduced as follows:

(9) $${\sigma =C}_{1}+C_{2}\varepsilon^{n}$$

where $C_{1}$ represents the yield stress, which was measured as 498 MPa. Through regression analysis, the two constants $C_{2}$ and n were identified as 125.34 MPa and 0.51, respectively.

At 0.01 s^−1^, and after certain modifications, Equation (6) is written as follows:

(10) $$\ln\left[\frac{\sigma }{C_{1}+C_{2}\varepsilon^{n}}\right]=\left[-\left(C_{3}+C_{4}\varepsilon \right)\right]T^{*}$$

The constants $C_{3}$ and $C_{4}$ were calculated from the regression analysis as 0.0042 and 0.0066, respectively. By taking natural logarithms and after making several rearrangements, Equation (6) is written as described in Equation (11). The remaining values of $\dot{\varepsilon }$ and T were used to determine $C_{5}$ and $C_{6}$ using the regression analysis as 0.0042 and 0.0066, respectively. All the material constants included in the MZA model for AA2060 alloy were obtained as listed in Table 1.

(11) $$\ln\left[\frac{\sigma }{C_{1}+C_{2}\varepsilon^{n}}\right]+\left(C_{3}+C_{4}\varepsilon \right)T^{*}=\left(C_{5}+C_{6}T^{*}\right)\ln\varepsilon^{˙*}$$

Thus, the MZA constitutive model for AA2060 alloy can be written as follows:

(12) $$\sigma =\left(498+125.34\varepsilon^{0.51}\right)\exp\left[-\left(0.0042+0.0066\varepsilon \right)T^{*}+\left(0.0184+0.0001T^{*}\right)\ln\varepsilon^{˙*}\right]$$

**Figure 2:**
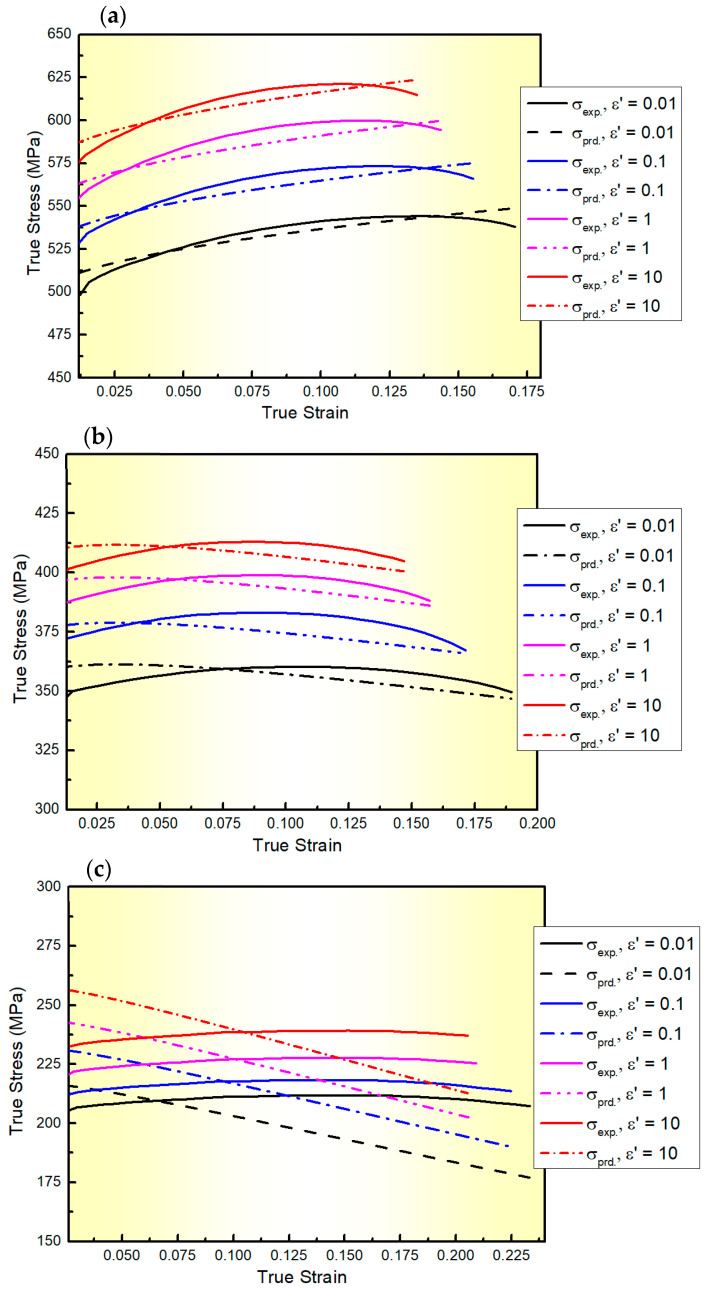

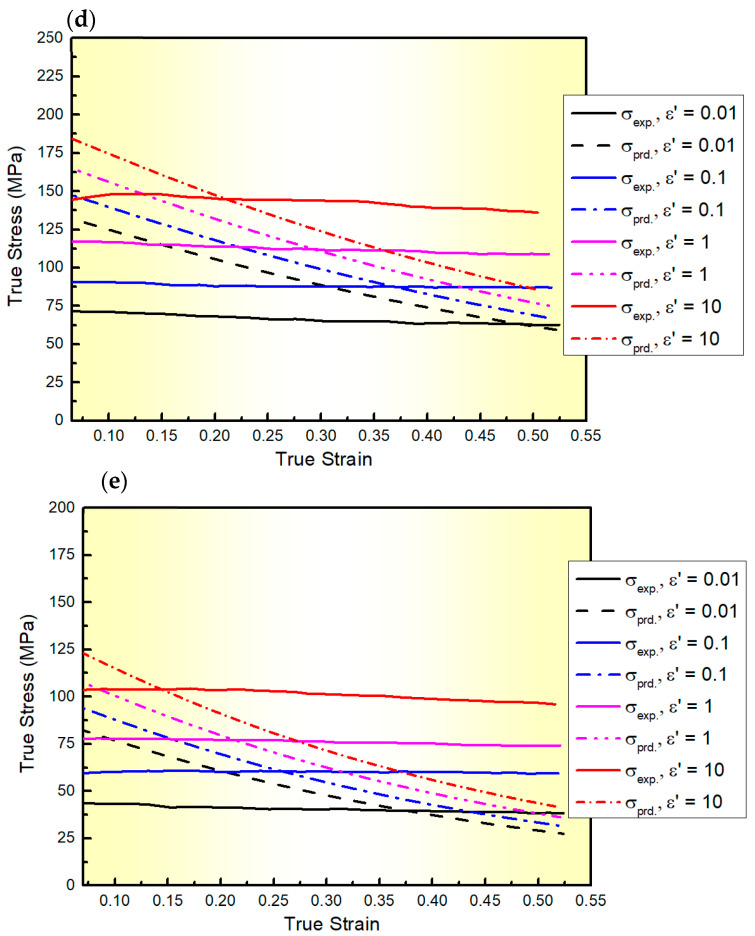
The flow curves of AA2060 sheets determined from the developed MZA model (dashed lines) and their counterparts acquired via experimentation (solid lines) at the $\dot{\varepsilon }$ range of 0.01–10 s^−1^ and testing temperatures of (**a**) 100, (**b**) 200, (**c**) 300, (**d**) 400, and (**e**) 500 °C, respectively.

The comparison between the flow stresses determined from the MZA model and their counterparts acquired from experimentation is depicted in Figure 2. As shown in this figure, the MZA model demonstrates moderate accuracy in predicting the flow behaviour of the AA2060 alloy sheet at elevated temperatures across all tested $\dot{\varepsilon }$, particularly at or near the reference $\dot{\varepsilon }$ and T. This accuracy is attributed to the model’s incorporation of thermal softening, strain-rate hardening, isotropic hardening, and the combined effects of ε, $\dot{\varepsilon }$, and T on flow behaviour. Similar results have been observed in metallic materials such as 316L, 304 stainless steels, and 9Cr–1Mo alloy steel [35].

Despite the visual assessment, the reliability and predictability of the MZA model were further assessed by computing standard statistical parameters. These parameters include the correlation coefficient (*R*), average absolute relative error (*AARE*), and root mean square error (*RMSE*). *R* is a vital statistical parameter in providing information on the reliability and accuracy of the linear relationships between the predicted and the experimental values. However, since *R* was determined by a point-by-point comparison of relative errors, *AARE* is regarded as an unbiased parameter for assessing the accuracy and reliability of developed models. The small values of *AARE* indicate a higher level of predictability in the developed model and vice versa. The calculations of *R*, *AARE*, and *RMSE* were carried out using Equations (13)–(15), respectively.

(13) $$R=\frac{\sum_{i=1}^{i=N}\left(\sigma_{E.}^{i}-{\bar{\sigma }}_{E})(\sigma_{P}^{i}-{\bar{\sigma }}_{P}\right)}{\sqrt{\sum_{i=1}^{i=N}{\left(\sigma_{E}^{i}-{\bar{\sigma }}_{E}\right)}^{2}\sum_{i=1}^{i=N}{\left(\sigma_{P}^{i}-{\bar{\sigma }}_{P}\right)}^{2}}}$$

(14) $$AARE\;(\%)=\frac{1}{N}\;\sum_{i=1}^{i=N}\left|\frac{\sigma_{E\;}^{i}-\sigma_{P}^{i}}{\sigma_{E.\;}^{i}}\right|\times 100$$

(15) $$RMSE=\sqrt{\frac{1}{N}\sum_{i=1}^{i=N}{\left(\sigma_{E}^{i}-\sigma_{P}^{i}\right)}^{2}}$$

where *N* represents the total number of points included in the analysis. $\sigma_{E}^{i}$ and $\sigma_{P}^{i}$ denote the experimental and predicted stress values, respectively. ${\bar{\sigma }}_{E}$ and ${\bar{\sigma }}_{P}$ are the mean values of the experimental and the predicted stress values, respectively. The *R*, *AARE*, and *RMSE* values of the MZA model for the AA2060 alloy are 0.984, 13.67%, and 21.58 MPa, respectively.

#### 3.2.2. Lin’s Modified Johnson–Cook Model (L-MJC)

Lin et al. [50] developed the original JC model and proposed their model, which is named L-MJC. In this modification, they considered the interaction between $\dot{\varepsilon }$ and T very carefully. The L-MJC is described as follows:

(16) $$\sigma =\left(A+{B_{1}\varepsilon +B_{2}\varepsilon }^{2}\right)\left(1+C_{1}\ln\varepsilon^{˙*}\right)\exp\left[\left(\lambda_{1}+\lambda_{2}\ln\varepsilon^{˙*}\right)\left(T-T_{r}\right)\right]$$

where σ and ε are the flow stress and plastic strain, respectively. $\lambda_{2}$, $\lambda_{1}$, $C_{1},\;\;B_{2},\;{\;B}_{1},$ and A, are the material constants. $\varepsilon^{˙*}$ is the ratio between the experimental and reference $\dot{\varepsilon }$, which is described by Equation (7). Furthermore, T describes the testing temperature and $T_{r}$ represents the reference temperature.

To determine the L-MJC model constants in this investigation, ${\dot{\varepsilon }}_{r}$ and $T_{r}$ are adjusted to be 0.01 s^−1^ and 100 °C, respectively. Thus, Equation (16) is reduced as follows:

(17) $$\sigma =A+{B_{1}\varepsilon +B_{2}\varepsilon }^{2}$$

Through regression analysis, the constants $A,\;{\;B}_{1}$, and $B_{2}$ were determined to be 494.13, 784.69, and −3069.85 MPa.

At 100 °C, and after completing some adjustments, Equation (16) was written as follows:

(18) $$\frac{\sigma }{A+{B_{1}\varepsilon +B_{2}\varepsilon }^{2}}-1=C_{1}\ln\varepsilon^{˙*}$$

By using the regression analysis, the constant $C_{1}$ is computed through regression analysis as 0.0221. After taking the natural logarithm and making several adjustments at different $\dot{\varepsilon }$, Equation (16) can be expressed as described in Equation (19). $\lambda_{1}$ and $\lambda_{2}$ are two constants calculated using regression analysis as −0.0048 and 0.0001, respectively. The determined constants of L-MJC for the AA2060 alloy are listed in Table 2.

(19) $$\frac{\ln\left[\frac{\sigma }{\left(A+{B_{1}\varepsilon +B_{2}\varepsilon }^{2}\right)\left(1+C_{1}\ln\varepsilon^{˙*}\right)}\right]}{T-T_{r}}=\lambda_{1}+\lambda_{2}\ln\varepsilon^{˙*}$$

Thus, the L-MJC constitutive model for the AA2060 alloy can be written as follows:

(20) $$\sigma =\left(494.13+{784.69\varepsilon -3069.85\varepsilon }^{2}\right)\left(1+0.0221\ln\varepsilon^{˙*}\right)\exp\left[\left(-0.0048+0.0001\ln\varepsilon^{˙*}\right)\left(T-T_{r}\right)\right]$$

**Figure 3:**
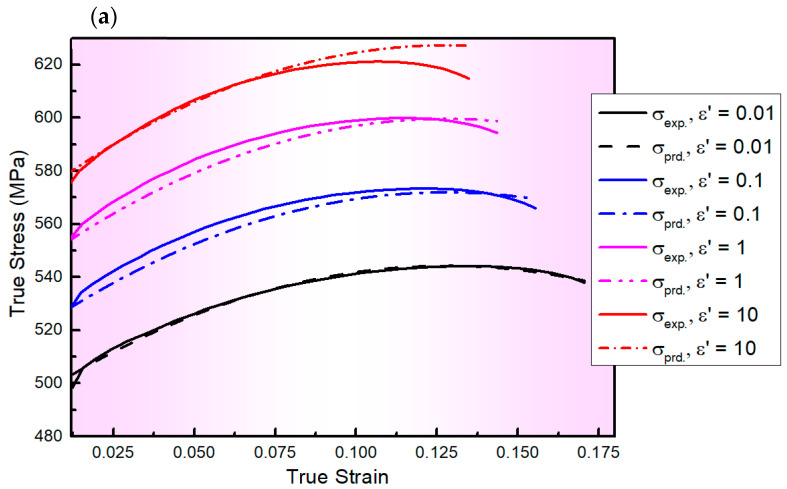

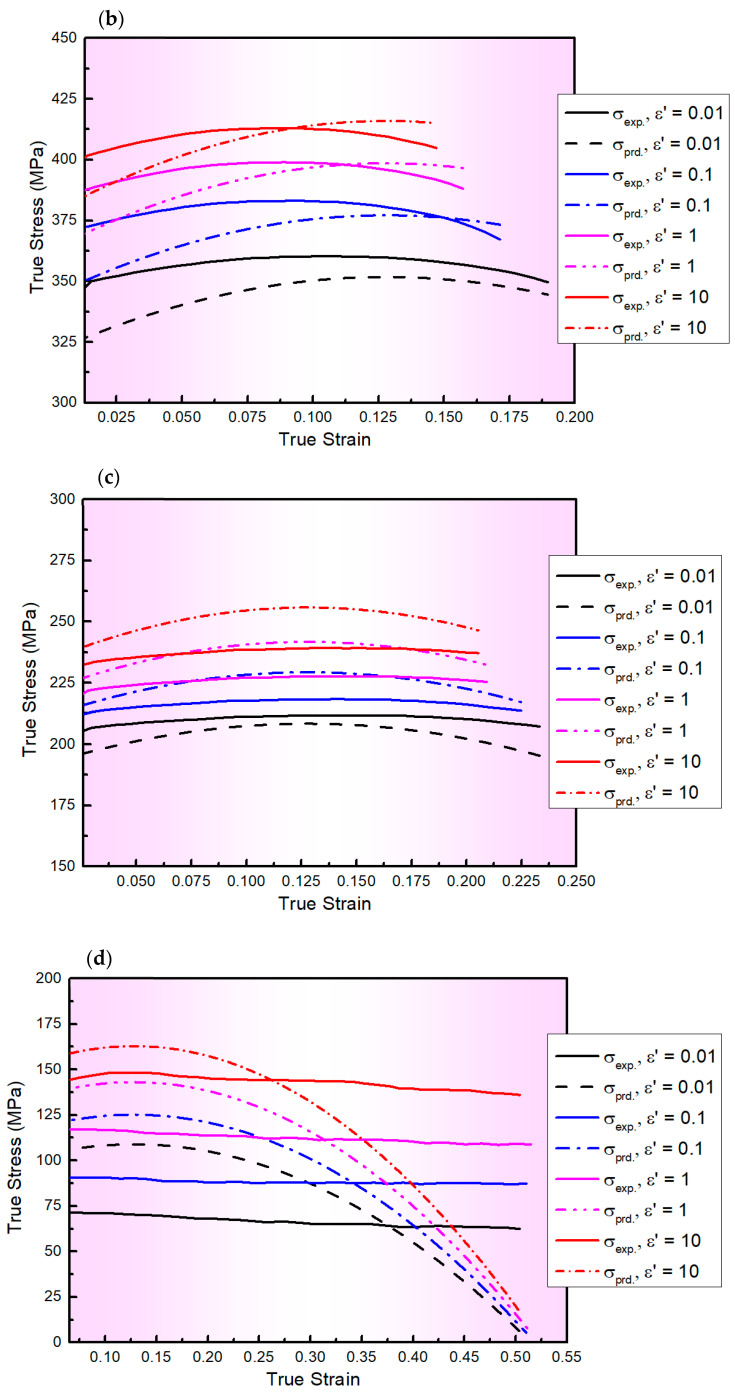

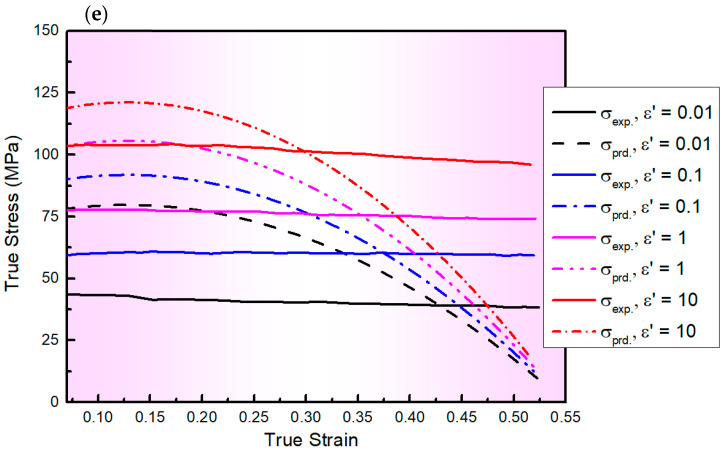
The flow curves of AA2060 sheets predicted from the developed L-MJC model (dashed lines) and their counterparts acquired via experimentation (solid lines) at the $\dot{\varepsilon }$ range of 0.01–10 s^−1^ and testing temperatures of (**a**) 100, (**b**) 200, (**c**) 300, (**d**) 400 and (**e**) 500 °C, respectively.

Figure 3 displays a comparison between the experimental stress values and those predicted by the L-MJC model for AA2060 alloy at a wide range of $\dot{\varepsilon }$ and T with *R*, *AARE*, and *RMSE* values (calculated using Equations (14)–(16)) of 0.979, 17.38%, and 26.17 MPa, respectively. As noticed from Figure 3 and confirmed by calculating *R*, *AARE*, and *RMSE*, the L-MJC model does not accurately predict the flow behaviour of AA2060 alloy under these conditions. This may be because L-MJC only focuses on the interaction between $\dot{\varepsilon }$ and T, while neglecting the interactions between ε and the combined effects of $\dot{\varepsilon }$ and T.

#### 3.2.3. Shokry’s Modified Johnson–Cook Model-1 (S1-MJC)

Shokry [51] modified the original JC model to accurately determine the flow behaviour of 800 H alloy under high T and intermediate $\dot{\varepsilon }$, by linking the ε directly with both $\dot{\varepsilon }$ and T using a linear relationship. The S1-MJC model is written as follows:

(21) $$\sigma =\left(A+{B_{1}\varepsilon +B_{2}\varepsilon }^{2}+{B_{3}\varepsilon }^{3}\right)\left(1+\left(C_{1}+C_{2}\varepsilon \right)\ln\varepsilon^{˙*}\right)\left(1-{T^{*}}^{\left(m_{1}+m_{2}\varepsilon \right)}\right)$$

where σ and ε are the flow stress and plastic strain, respectively. Furthermore, $m_{2}$, $m_{1},\;{\;C}_{2},\;{\;C}_{1},{\;B}_{3},{\;B}_{2},{\;B}_{1},$ and A are material constants. $\varepsilon^{˙*}$ is the ratio between the experimental and reference of $\dot{\varepsilon }$, which is described by Equation (7). $T^{*}$ is presented as described in Equation (22), where T is the testing temperature, $T_{m}$ represents the melting temperature of AA2060, and $T_{r}$ is the reference temperature.

(22) $$T^{*}=\frac{T-T_{r}}{T_{m}-T_{r}}$$

To determine the S1-MJC model constants in this investigation, ${\dot{\varepsilon }}_{r}$ and $T_{r}$ are adjusted to be 0.01 s^−1^ and 100 °C, respectively. Therefore, Equation (21) is simplified as follows:

(23) $$\sigma =A+{B_{1}\varepsilon +B_{2}\varepsilon }^{2}+{B_{3}\varepsilon }^{3}$$

By using the regression analysis, the constants $A,\;{\;B}_{1},\;{\;B}_{2}$ and $B_{3}$ were calculated as 492.31, 883.74, −4376.80, and 4798.92 MPa, respectively.

At 100 °C, and after simplification, Equation (21) was described as follows:

(24) $$\frac{\sigma }{A+{B_{1}\varepsilon +B_{2}\varepsilon }^{2}+{B_{3}\varepsilon }^{3}}-1=\left(C_{1}+C_{2}\varepsilon \right)\ln\varepsilon^{˙*}$$

The constants $C_{1}$ and $C_{2}$ were calculated through the regression analysis as 0.0244 and −0.0189, respectively.

After taking the natural logarithm and performing simplifications, and at different $\dot{\varepsilon }$ values, Equation (21) can be expressed as follows:

(25) $$\ln\left[1-\frac{\sigma }{\left(A+{B_{1}\varepsilon +B_{2}\varepsilon }^{2}+{B_{3}\varepsilon }^{3}\right)\left(1+\left(C_{1}+C_{2}\varepsilon \right)\ln\varepsilon^{˙*}\right)}\right]=\left(m_{1}+m_{2}\varepsilon \right){\;T}^{*}$$

where $m_{2}$ and $m_{1}$ are constants computed through the regression analysis as −0.1791 and 0.5717. The constants of S1-MJC for the AA2060 alloy are written in Table 3.

Thus, the S1-MJC constitutive model for the AA2060 alloy can be written as follows:

(26) $$\sigma =\left(492.31+{883.74\varepsilon -4376.80\varepsilon }^{2}+{4798.92\varepsilon }^{3}\right)\left(1+\left(0.0244-0.0189\varepsilon \right)\ln\varepsilon^{˙*}\right)\left(1-{T^{*}}^{\left(0.5717-0.1791\varepsilon \right)}\right)\;$$

**Figure 4:**
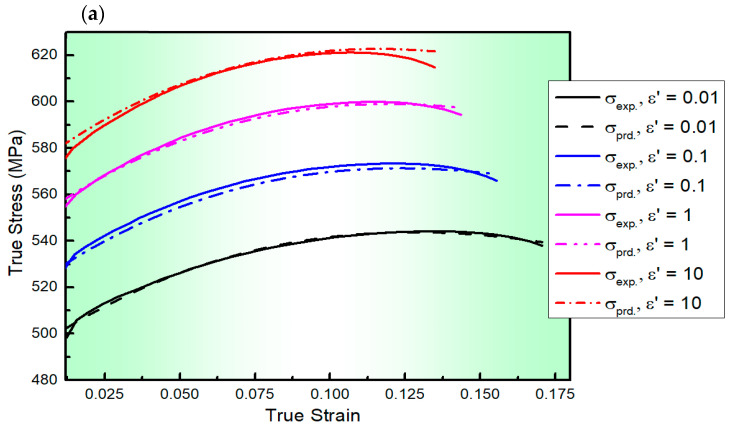

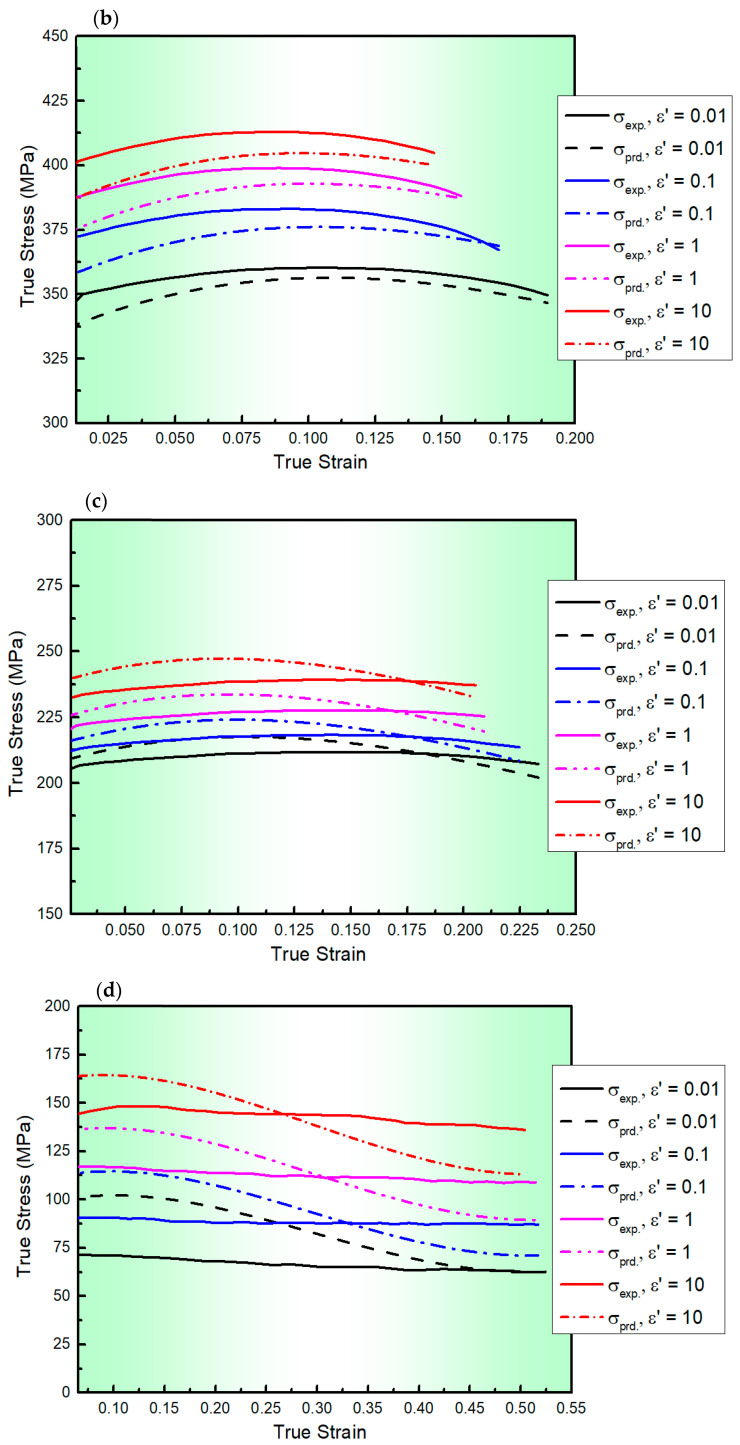

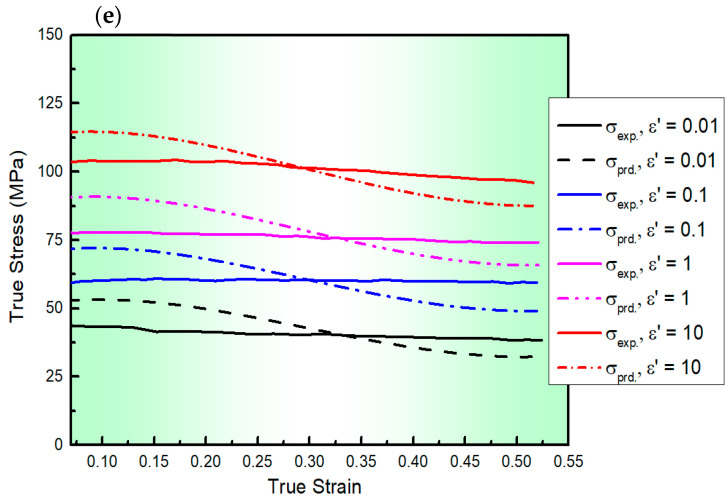
The flow curves of AA2060 sheets obtained from the developed S1-MJC model (dashed lines) and their counterparts acquired via experimentation (solid lines) at the $\dot{\varepsilon }$ range of 0.01–10 s^−1^ and testing temperatures of (**a**) 100, (**b**) 200, (**c**) 300, (**d**) 400, and (**e**) 500 °C, respectively.

Figure 4 depicts a comparison between the values of experimental and predicted stresses determined by the S1-MJC model for the AA2060 alloy at a wide range of $\dot{\varepsilon }$ and T with *R*, *AARE*, and *RMSE* values of 0.988, 10.47%, and 17.08 MPa, respectively. As observed from Figure 4 and verified by calculating *R*, *AARE*, and *RMSE*, the predicted results obtained by the S1-MJC model fit well with their counterparts acquired from experimentation, demonstrating a better fit compared to the L-MJC model. This is because of the linking impact found between both softening and of $\dot{\varepsilon }$ and strain hardening in the S1-MJC model. It is widely known that the dislocation processes are affected by softening and of $\dot{\varepsilon }$.

#### 3.2.4. Shokry’s Modified Johnson–Cook Model-2 (S2-MJC)

In a recent study, Shokry et al. [52] proposed a modified generic JC model named S2-MJC to predict the hot flow behaviour of several metallic alloys. Their model is expressed as follows:

(27) $$\sigma =\left(\sum_{i=0}^{3}A_{i}\varepsilon^{i}\right)\left(1+\left(\sum_{i=0}^{2}\sum_{j=0}^{2}C_{ij\;}\varepsilon^{i}\varepsilon^{˙j}\right)\ln\varepsilon^{˙*}\right)\exp\left[\left(\sum_{i=0}^{2}\sum_{j=0}^{2}\sum_{k=0}^{2}{m_{ijk}\;\varepsilon }^{i}\varepsilon^{˙j}T^{*k}\right)T^{*}\right]$$

where $\sigma ,\;\;\varepsilon ,\;{\;\varepsilon }^{˙},\;{\;\varepsilon }^{˙*}$, and $T^{*}$ were defined above in the S1-MJC constitutive model. The $A_{i}$ constants correlate with the $\varepsilon^{i}$ strain, which represents the strain-hardening component. The $C_{ij\;}$ constants associate with the $\varepsilon^{˙*}$, while the $m_{ijk}$ constants relate to the softening parameter $T^{*}$. In this investigation, ${\dot{\varepsilon }}_{r}$ and $T_{r}$ are set to 0.01 s^−1^ and 100 °C, respectively, to obtain the constants of the S2-MJC model. Hence, Equation (27) is reduced as follows:

(28) $$\sigma =\sum_{i=0}^{3}A_{i}\varepsilon^{i}$$

After expanding Equation (28), it is extended into four terms involving ε, each accompanied by four constants. These constants are determined via regression analysis to be 492.31, 883.74, −4376.80, and 4798.92 MPa, respectively.

At 100 °C, and after some adjustments, (Equation (27)) is written as follows:

(29) $$\left(\frac{\sigma }{\sum_{i=0}^{3}A_{i}\varepsilon^{i}}-1\right)/\ln\varepsilon^{˙*}=\sum_{i=0}^{2}\sum_{j=0}^{2}C_{ij\;}\varepsilon^{i}\varepsilon^{˙j}$$

After expanding Equation (29), nine terms including ε and $\dot{\varepsilon }$ were determined. These nine terms are associated with nine constants determined through regression analysis. The constants are calculated to be 0.0245, −0.0013, 0.0001, 0.0471, −0.0188, 0.0018, −0.4575, 0.1516, and −0.0145.

After taking the natural logarithm and making several adjustments for various $\dot{\varepsilon }$, Equation (27) can be written as Equation (30).

(30) $$\frac{\ln\left[\frac{\sigma }{\left(\sum_{i=0}^{3}A_{i}\varepsilon^{i}\right)\left(1+\left(\sum_{i=0}^{2}\sum_{j=0}^{2}C_{ij\;}\varepsilon^{i}\varepsilon^{˙j}\right)\ln\varepsilon^{˙*}\right)}\right]}{T^{*}}=\sum_{i=0}^{2}\sum_{j=0}^{2}\sum_{k=0}^{2}{m_{ijk}\;\varepsilon }^{i}\varepsilon^{˙j}T^{*k}$$

The right-hand side of the equation is expanded and includes 27 terms involving ε, $\dot{\varepsilon }$, and T. Each term of the 27 constants was determined through regression analysis: −1.1437, 0.0565, −0.0062, −8.3062, −0.4676, 0.0633, 47.2185, −9.5081, 0.6546, −3.2713, −0.6143, 0.0571, 10.8928, 6.2511, −0.5799, −96.990, 24.966, −1.7706, 0.8372, 2.3817, −0.2075, 1.4364, −11.601, 1.0106, 61.103, −15.1629, and 1.0568. The calculated constants of the S2-MJC model for the AA2060 alloy are listed in Table 4.

**Figure 5:**
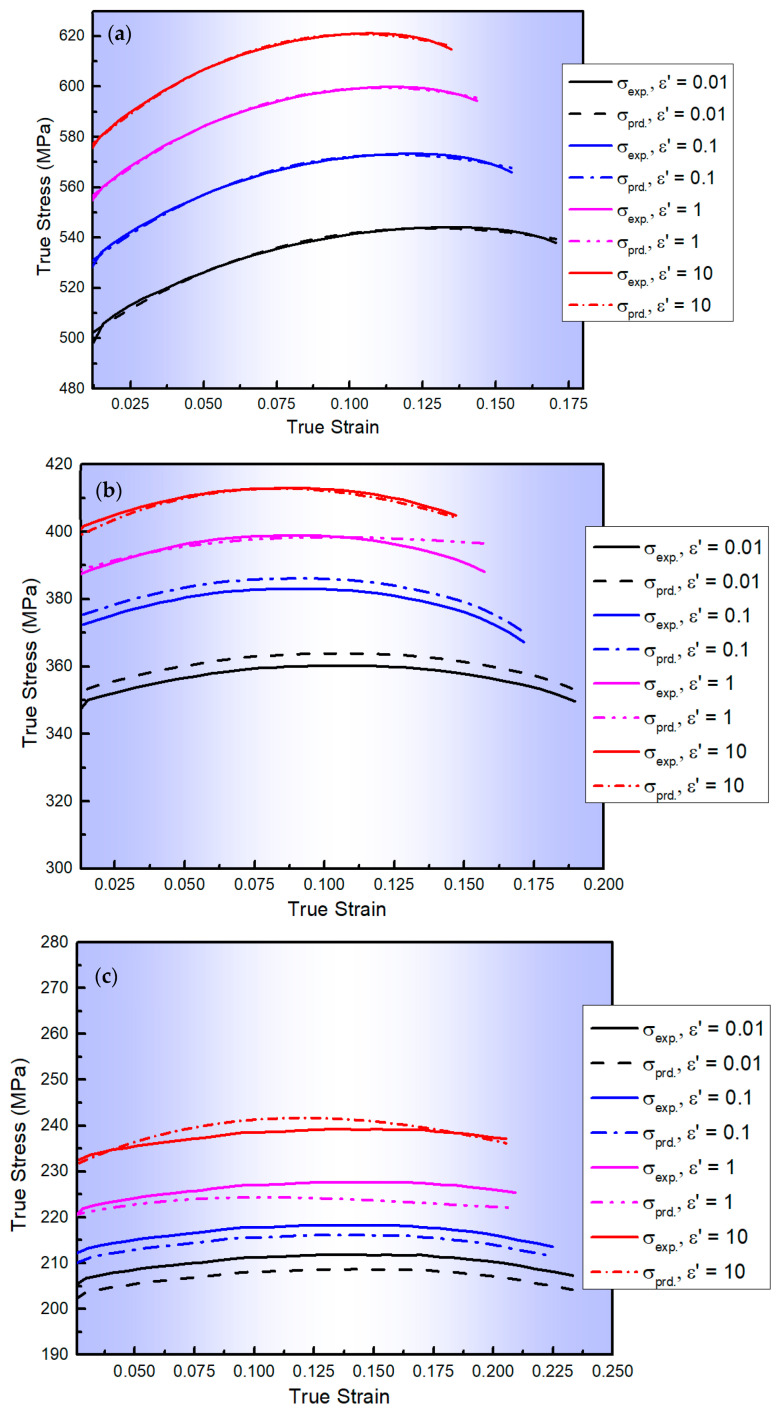

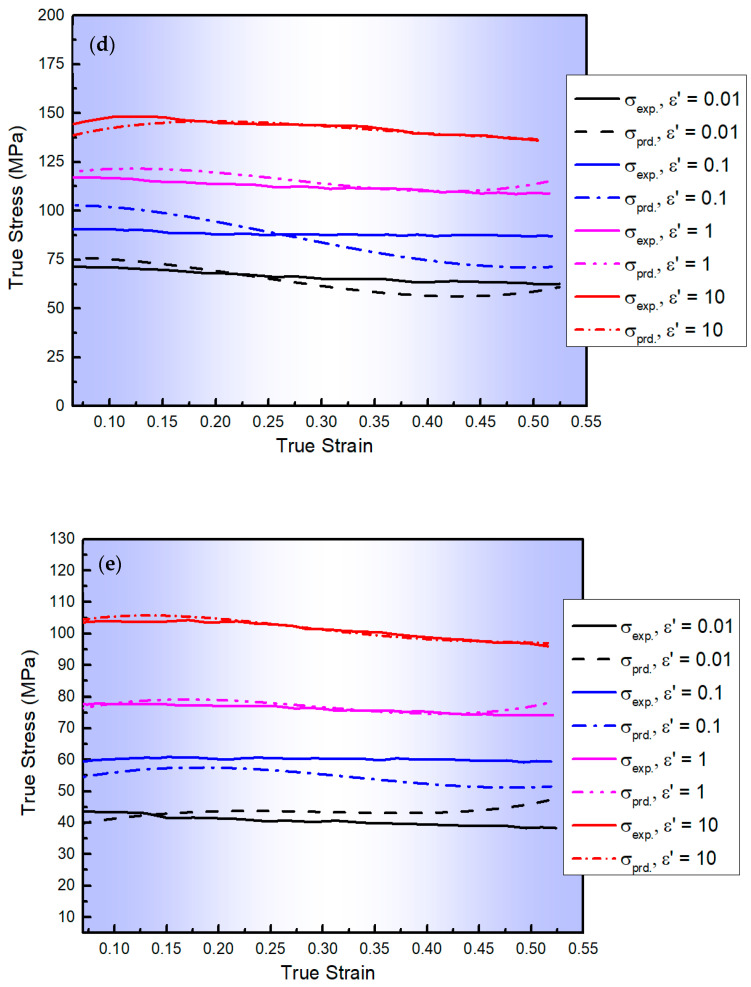
The flow curves of AA2060 sheets determined from the developed S2-MJC model (dashed lines) and their counterparts acquired via experimentation (solid lines) at the $\dot{\varepsilon }$ range of 0.01–10 s^−1^ and testing temperatures of (**a**) 100, (**b**) 200, (**c**) 300, (**d**) 400, and (**e**) 500 °C, respectively.

**Figure 6:**
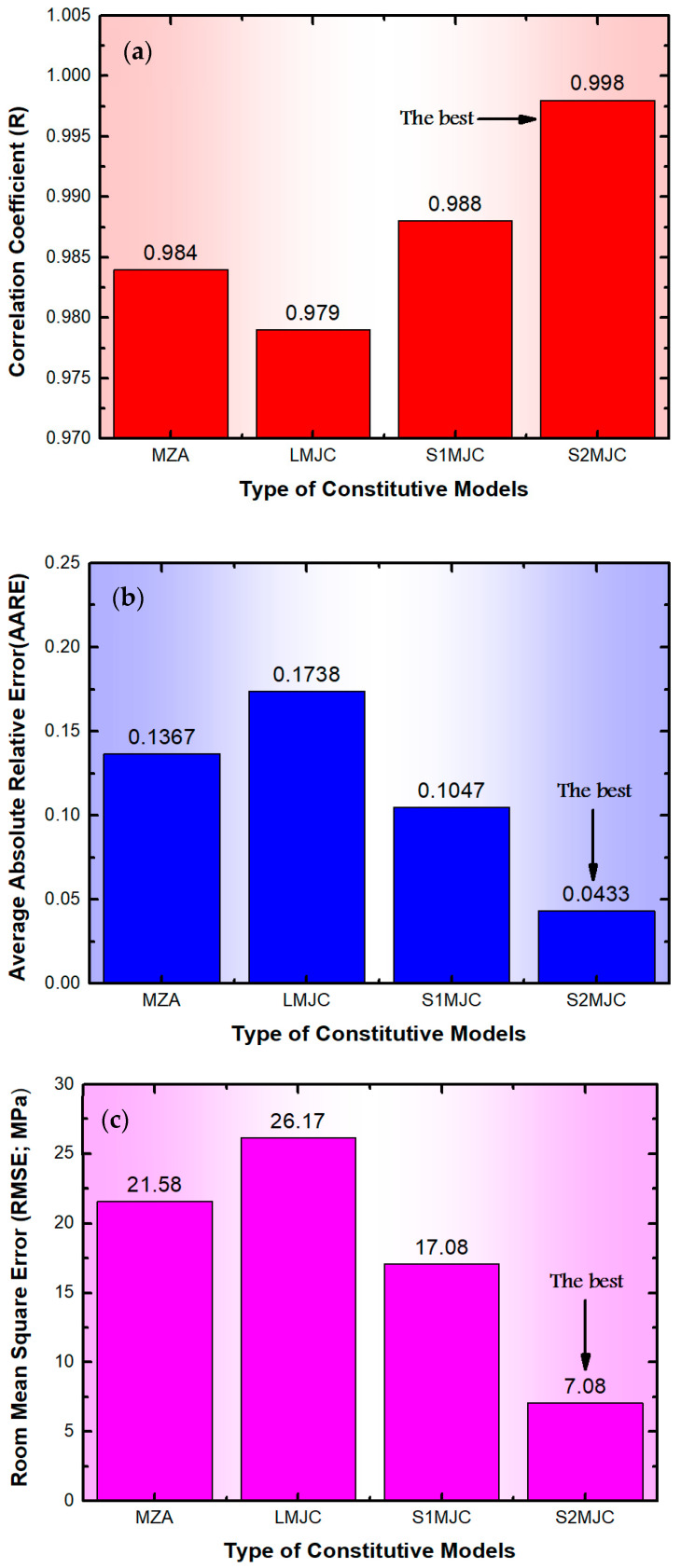
A comparison between (**a**) R, (**b**) AARE, and (**c**) RMSE of the MZA, L-MJC, S1-MJC, and S2-MJC constitutive models to verify the predictability.

Figure 5 presents a comparison between the values of predicted stresses determined by the S2-MJC model for the AA2060 alloy at a wide range of $\dot{\varepsilon }$ and T and their experimental counterparts. Furthermore, the statistical parameters *R*, *AARE*, and *RMSE* were calculated to be 0.999, 4.33%, and 7.08 MPa, respectively. As evident from Figure 5 and confirmed by the calculations of *R*, *AARE*, and *RMSE* as depicted in Figure 6, the values of the predicted stresses determined using the S2-MJC model align closely with the experimental stresses, demonstrating a remarkable fit compared to the S1-MJC, L-MJC, and MZA models. This is because of the linking impact between softening, the strain rate, and strain hardening in the S2-MJC model. It is widely known that the dislocation process is affected by softening and strain rates. This is attributed to the interactions that occurred between ε and $\dot{\varepsilon }$ from one side and between ε, $\dot{\varepsilon }$, and T from the other side using an extensive set of constants correlating the constitutive components of dynamic recovery and softening mechanisms.

## 4. Conclusions

Based on the results obtained in this investigation, the main conclusions can be summarized as follows:

The MZA constitutive model for the AA2060 alloy was developed in this study as follows: $\sigma =\left(498+125.34\varepsilon^{0.51}\right)\exp\left[-\left(0.0042+0.0066\varepsilon \right)T^{*}+\left(0.0184+0.0001T^{*}\right)\ln\varepsilon^{˙*}\right]$. The MZA model demonstrated moderate accuracy in predicting the flow behaviour of the AA2060 alloy sheet across all tested conditions, particularly at or near the reference $\dot{\varepsilon }$ and T with *R*, *AARE*, and *RMSE* values of 0.984, 13.67%, and 121.58 MPa, respectively. This accuracy is attributed to the model’s incorporation of thermal softening, strain-rate hardening, isotropic hardening, and the combined effects of ε, $\dot{\varepsilon }$, and T.

The L-MJC constitutive model for the AA2060 alloy was developed in this study as follows:$\sigma =\left(494.13+{784.69\varepsilon -3069.85\varepsilon }^{2}\right)\left(1+0.0221\ln\varepsilon^{˙*}\right)\exp\left[\left(-0.0048+0.0001\ln\varepsilon^{˙*}\right)\left(T-T_{r}\right)\right]$. The L-MJC model does not accurately predict the flow behaviour of the AA2060 alloy across all tested conditions with *R*, *AARE*, and *RMSE* values of 0.979, 17.38%, and 26.17 MPa, respectively. This may be because L-MJC only focuses on the interaction between $\dot{\varepsilon }$ and T, while neglecting the interactions between ε and the combined effects of $\dot{\varepsilon }$ and T.

The S1-MJC constitutive model for the AA2060 alloy was developed in this study as follows: $\sigma =\left(492.31+{883.74\varepsilon -4376.80\varepsilon }^{2}+{4798.92\varepsilon }^{3}\right)\left(1+\left(0.0244-0.0189\varepsilon \right)\ln\varepsilon^{˙*}\right)\left(1-{T^{*}}^{\left(0.5717-0.1791\varepsilon \right)}\right).$ The predicted results obtained by the S1-MJC model fit well with those acquired from experimentation, demonstrating a better fit with the *R*, *AARE*, and *RMSE* values of 0.988, 10.47%, and 17.08 MPa, respectively, compared to the L-MJC model. This is because of the linking impact found between both softening of $\dot{\varepsilon }$ and strain hardening in the S1-MJC model. It is widely known that the dislocation processes are affected by softening and $\dot{\varepsilon }$.

The S2-MJC constitutive model for the AA2060 alloy was developed in this study as follows: $\frac{\ln\left[\frac{\sigma }{\left(\sum_{i=0}^{3}A_{i}\varepsilon^{i}\right)\left(1+\left(\sum_{i=0}^{2}\sum_{j=0}^{2}C_{ij}\varepsilon^{i}\varepsilon^{˙j}\right)\ln\varepsilon^{˙*}\right)}\right]}{T^{*}}=\sum_{i=0}^{2}\sum_{j=0}^{2}\sum_{k=0}^{2}{m_{ijk}\varepsilon }^{i}\varepsilon^{˙j}T^{*k}$. The right-hand side of the constitutive model is expanded and includes 27 terms involving ε, $\dot{\varepsilon }$, and T as listed in Table 4. The predicted stresses determined by the S2-MJC model align closely with the experimental stresses, and *R*, *AARE*, and *RMSE* were calculated to be 0.999, 4.33%, and 7.08 MPa, respectively, demonstrating a remarkable fit compared to the S1-MJC, L-MJC, and MZA models. This is because of the linking impact between softening, the strain rate, and strain hardening in the S2-MJC model. It is widely known that the dislocation process is affected by softening and strain rates. This is attributed to the interactions that occurred between ε and $\dot{\varepsilon }$ from one side and between ε, $\dot{\varepsilon }$, and T from the other side using an extensive set of constants correlating the constitutive components of dynamic recovery and softening mechanisms.

## Figures and Tables

**Table 1:** The constants of the MZA model for the AA2060 alloy.

$C_{1}[MPa]$	$C_{2}[MPa]$	n	$C_{3}$	$C_{4}$	$C_{5}$	$C_{6}$
498	125.34	0.51	0.0042	0.0066	0.0184	0.0001

**Table 2:** The constants of the L-MJC model for the AA2060 alloy.

A[MPa]	$B_{1}[MPa]$	$B_{2}[MPa]$	$C_{1}$	$\lambda_{1}$	$\lambda_{2}$
494.13	784.69	−3069.85	0.0221	−0.0048	0.0001

**Table 3:** The constants of the S1-MJC model for the AA2060 alloy.

A[MPa]	$B_{1}[MPa]$	$B_{2}[MPa]$	$B_{3}[MPa]$	$C_{1}$	$C_{2}$	$m_{1}$	$m_{2}$
492.31	883.74	−4376.80	4798.92	0.0244	−0.0189	0.5717	−0.1791

**Table 4:** The constants of the S2-MJC model for the AA2060 alloy.

$A_{0}$ [MPa]	$A_{1}$ [MPa]	$A_{2}$ [MPa]	$A_{3}$ [MPa]	$C_{00}$	$C_{01}$	$C_{02}$	$C_{10}$
492.31	883.74	−4376.8	4798.92	0.0245	−0.0013	0.0001	0.0471
$C_{11}$	$C_{12}$	$C_{20}$	$C_{21}$	$C_{22}$	$m_{000}$	$m_{001}$	$m_{002}$
−0.0188	0.0018	−0.4575	0.1516	−0.0145	−1.1437	0.0565	−0.0062
***m*_010_**	***m*_011_**	***m*_012_**	***m*_020_**	***m*_021_**	***m*_022_**	***m*_100_**	***m*_101_**
−8.3062	−0.4676	0.0633	47.2185	−9.5081	0.6546	−3.2713	−0.6143
$m_{102}$	$m_{110}$	$m_{111}$	$m_{112}$	$m_{120}$	$m_{121}$	$m_{122}$	$m_{200}$
0.0571	10.8928	6.2511	−0.5799	−96.990	24.966	−1.7706	0.8372
$m_{201}$	$m_{202}$	$m_{210}$	$m_{211}$	$m_{212}$	$m_{220}$	$m_{221}$	$m_{222}$
2.3817	−0.2075	1.4364	−11.601	1.0106	61.103	−15.1629	1.0568

## Data Availability

Data will be available upon request through the corresponding author due to the privacy, and the restrictions from the funding source.
